# Synthesis and Antimicrobial Screening of Pyrazolo-3-Aryl Quinazolin-4(3H)ones

**DOI:** 10.4103/0250-474X.73934

**Published:** 2010

**Authors:** M. B. Deshmukh, S. Patil, S. S. Patil, S. D. Jadhav

**Affiliations:** Organic Research Laboratory, Department of Chemistry, P. D. V. P. College, Tasgaon, Sangli 416312, India

**Keywords:** Acetic anhydride, hydrazino, hydrazones, pyrazolo-quinazolinones, quinazolinones

## Abstract

2-thio-3-aryl quinazolin-4(3H)one (1) was synthesized by reacting anthranilic acid with thiocarbamate salts of substituted aniline and carbon disulphide, which on reflux with excess of hydrazine hydrate to form 2-hydrazino quinazolin-4(3H)one derivatives (2). The reaction of (2) with variously substituted aryl aldehydes gave the corresponding hydrazones (3). Further, the cyclization of compound (3) in acetic anhydride gave tricyclic pyrazoloquinazolinones (4). All newly synthesized compounds have been tested for their antibacterial activity against gram +ve bacteria *B. substilis, S. aureus* and gram –ve bacteria *E. coli, P. vulgaris*. The species used for antifungal activity are *Aspergillus* niger and *Phytophora*. Introduction of -OCH3, -OH and -Cl groups to the heterocyclic frame work enhanced antibacterial and antifungal activities.

Derivatives quinazolines are of special importance because of their versatile biological activities[1,2], especially antihistaminic[3], antiinflammatory[4], antihypertensive[5], antiHIV[6], antifungal[6,7], antimicrobial[8,9], anticonvulsant[10], antithrombotic[11], antitubercular[12,13], antitumor[14], analgesic[15], antibacterial[6,15] and insecticidal[16]. In this paper, a new route for the synthesis of pyrazolo quinazolinones is reported.

The strategy employed for the synthesis of desired compounds involved the sequential treatment of anthranilic acid with thiocarbamate salts of substituted aniline and carbon disulphide to give substituted 2-thio-3-aryl quinazol-4(3H)ones (1). The appearance of broad band at 3330-3110 cm^-1^ in IR spectrum and a singlet displayed at δ, 10-13 ppm in the PMR spectrum due to -SH supports their formation. Compound (1) were refluxed with excess hydrazine hydrate to form 2-hydrazino derivative (2), the formation witch has been explained by the appearance of IR band at 3390-3100 cm^-1^ due to -NHNH_2_ and disappearance of signal observed at δ, 10-13 ppm due to -SH and the appearance of two additional singlet between δ, 9-11 and δ, 2-7 ppm due to -NH and -NH_2_ protons, respectively in their PMR spectra. The condensation of (2) with variously substituted aryl aldehydes gave the corresponding hydrazones (3). The appearance -NH and =CH protons at δ, 5.1 and δ, 8.2 in PMR spectrum and also disappearance of -NH_2_ band 3390-3200 cm^-1^ in IR spectrum indicated their formation. Further, the cyclization of compound (3) in acetic anhydride gave tricyclic triazolo quinazolones (4). The formation of these compounds have been established by the disappearance of the PMR singlet due to -NH and =CH displayed at δ, 5.1 and δ, 8.2, respectively in the PMR spectrum of (3).(Scheme 1)

**Scheme 1:**
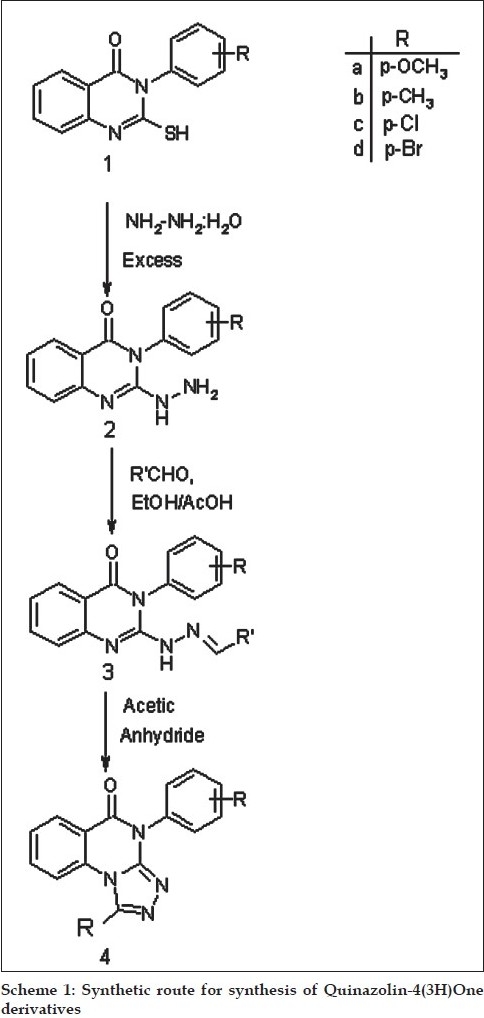
Synthetic route for synthesis of Quinazolin-4(3H)One derivatives

All chemicals used were of AR grade and are used without further purification. Melting points were determined by open capillary method and are uncorrected. ^1^H NMR spectra in DMSO-d_6_ were scanned on a Bruker A-300 F-NMR spectrometer. IR spectra were recorded on a Perkin-Elmer 783 (FTIR) spectrophotometer. Purity of the products in addition to the elemental analysis was checked by TLC.

The starting compound (1) was prepared by reported method[17]. 2-Hydrazino 3-p-methoxy phenyl quinazolin-4(3H)one (2a) was synthesized as follows; The compound (la) (5.0 g, 0.018 mole) was refluxed with excess of hydrazine hydrate (15 ml) with constant stirring at 100°C for about 1 ½ h, cooled and the solid obtained was filtered and recrystallized from ethanol to furnish (2a), IR(KBr); 3386-3328 (-HNNH _2_), 1664 (cyclic amido C=O) cm^-1^; ^1^H NMR (DMSO-d_6_): δ, 3.81(3H,s,Ar-OCH_3_), 6.25(2H,s,-NH_2_), 6.9-7.8(8H,m, Ar-H), 10.9(1H,s, br, -NH).

2-(p-Methoxybenzylidene)hydrazine-3-(p-methoxyphenyl)quinazolin-4-(3H)one (3a_-1_) was synthesized using following procedure; the mixture of compound (2a) (0.2 g, 0.0007 mole) and p-methoxy benzaldehyde (0.1 g, 0.0007 mole) in ethanol (10 ml) to which two drops of acetic acid were added and the reaction mixture heated on oil bath for 5 h. The separated solid was filtered under vacuum and further recrystallized from DMF, IR(KBr); 3100-3350(-NH), 1665 cm^-1^ (cyclic amido >C=O), 1600 cm^-1^(-C=N); ^1^H NMR (DMSO-d_6_): 3.82 (3H, s, Ar-OCH_3_), 3.84 (3H, s, another Ar-OCH_3_), 6.00 (1H, s, br, -NH), 8.21 (1H, s, =CH), 6.8-8.1 (12H, m, Ar-H), 8.20 (=CH) ppm.

3,5’-(p-Dimethoxyphenyl) pyrazolo-[3’,4’-a] quinazolin-4(3H)one (4_a-1_) was synthesized as follows; To a solution of compound (3_a-1_) (0.1 g, 0.00025 mole) in acetic anhydride (10 ml) was refluxed for about 2 h then poured in ice-cold water and separated solid was filtered, recrystallized from DMF to get desired tricyclic pyrazolo quinazolinones, IR (KBr): 1665 cm^-1^ (cyclic amido > C=O) and 1620 cm^-1^ (C=N); ^1^H NMR: (DMSO-d_6_): 3.79 (3H, s, Ar-OCH_3_) 3.95(3H, s, another Ar-OCH_3_), 6.8-8.2(12H, m, Ar-H) ppm. (Table 1)

**TABLE 1:** CHARACTERIZATION DATA OF COMPOUNDS 2, 3 AND 4

S. No.	R Groups	M.P.(o)	Yield (%)	Mol. Formula	% C		% H		% N	
					Cal.	Found	Cal.	Found	Cal.	Found
2a	p-OCH_3_	205	76	C_15_H_14_O_2_N_4_	63.82	63.76	5.00	5.10	19.85	19.90
2b	p-CH_3_	215	86	C_15_H_14_ON_4_	67.65	67.59	5.30	5.38	21.04	21.10
2c	p-Cl	218	82	C_14_H_11_ON_4_Cl	58.65	58.58	3.87	4.95	19.54	19.47
2d	p-Br	196	83	C_14_H_11_ON_4_Br	50.78	50.62	3.35	3.40	16.92	16.86
	R’groups									
4_a-1_	p-OCH_3_C_6_H_4_	230	83	C_23_H_18_O_3_N_4_	69.34	69.45	4.55	4.41	14.06	13.86
4_a-2_	o-NO_2_C_6_H_4_	193	78	C_22_H_15_O_4_N_5_	63.92	63.85	3.66	3.70	16.94	16.82
4_a-3_	3,4,5(OCH_3_)3-C_6_H_2_	211	68	C_25_H_22_O_5_N_4_	65.49	67.00	4.84	4.89	12.22	12.18
4_a-4_	o-OHC_6_H_4_	216	77	C_22_H_16_O_3_N_4_	68.74	68.80	4.20	4.16	14.58	14.49
4_a-5_	p-OHC_6_H_4_	239	71	C_22_H_16_O_3_N_4_	68.74	68.65	4.20	4.18	14.58	14.50
4_a-6_	o-ClC_6_H_4_	241	70	C_22_H_15_O_2_N_4_Cl	65.60	65.60	3.75	3.68	13.91	14.00
4_a-7_	p-ClC_6_H_4_	235	72	C_22_H_15_ON_4_Cl	65.60	65.58	3.75	3.81	13.91	13.86
4_a-8_	p-OH,m-OCH_3_-C_6_H_3_	243	78	C_23_H_18_O_4_N_4_	66.66	66.70	4.38	4.40	13.52	13.60
4_b-1_	p-OCH_3_C_6_H_4_	198	86	C_23_H_18_O_2_N_4_	72.24	72.30	4.74	4.81	14.65	14.72
4_b-2_	o-NO_2_C_6_H_4_	175	81	C_22_H_15_O_3_N_5_	66.49	66.40	3.80	3.75	17.62	17.70
4_b-3_	3,4,5(OCH_3_)3-C_6_H_2_	203	78	C_25_H_22_O_4_N_4_	67.86	67.70	5.01	5.11	12.66	12.73
4_b-4_	o-OHC_6_H_4_	218	68	C_22_H_16_O_2_N_4_	71.73	71.62	4.38	4.29	15.21	15.30
4_b-5_	p-OHC_6_H_4_	221	62	C_22_H_16_O_2_N_4_	71.73	71.66	4.38	4.30	15.21	15.16
4_b-6_	o-ClC_6_H_4_	216	72	C_22_H_15_ON_4_Cl	68.31	68.40	3.91	3.83	14.48	14.53
4_b-7_	p-ClC_6_H_4_	235	72	C_22_H_15_ON_4_Cl	68.31	68.39	3.91	4.00	14.48	14.51
4_b-8_	p-OH,m-OCH_3_-C_6_H_3_	235	81	C_23_H_18_O_3_N_4_	69.34	69.28	4.55	4.49	14.06	14.10
4_c-1_	p-OCH_3_C_6_H_4_	246	86	C_22_H_15_O_2_N_4_Cl	65.60	64.30	3.75	3.81	13.91	13.84
4_c-2_	o-NO_2_C_6_H_4_	198	82	C_21_H_12_O_3_N_5_Cl	60.37	60.45	2.89	2.81	16.76	16.68
4_c-3_	3,4,5(OCH_3_)3-C_6_H_2_	261	79	C_24_H_24_O_4_N_4_Cl	62.27	62.32	4.14	4.21	12.10	12.05
4_c-4_	o-OHC_6_H_4_	232	71	C_21_H_13_O_2_N_4_Cl	64.87	64.91	3.37	3.42	14.41	14.35
4_c-5_	p-OHC_6_H_4_	222	69	C_21_H_13_O_2_N_4_Cl	64.87	65.00	3.37	3.40	14.41	14.50
4_c-6_	o-ClC_6_H_4_	248	65	C_21_H_12_ON_4_Cl2	61.93	61.85	2.97	3.05	13.76	13.81
4_c-7_	p-ClC_6_H_4_	242	80	C_21_H_12_ON_4_Cl2	61.93	61.86	2.97	3.10	13.76	13.82
4_c-8_	p-OH,m-OCH_3_-C_6_H_3_	243	65	C_22_H_15_O_3_N_4_Cl	63.09	63.13	3.61	3.53	13.38	13.31
4_d-1_	p-OCH_3_C_6_H_4_	246	86	C_21_H_15_O_2_N_4_Br	59.08	59.10	3.38	3.42	12.53	12.60
4_d-2_	o-NO_2_C_6_H_4_	198	82	C_21_H_12_O_3_N_5_Br	54.56	54.50	2.62	2.56	15.15	15.10
4_d-3_	3,4,5(OCH_3_)-C_6_H_2_	261	79	C_24_H_19_O_4_N_4_Br	56.82	56.89	3.77	3.68	11.04	11.12
4_d-4_	o-OHC_6_H_4_	232	71	C_21_H_13_O_2_N_4_Br	58.22	58.16	3.02	3.11	12.93	12.84
4_d-5_	p-OHC_6_H_4_	222	69	C_21_H_13_O_2_N_4_Br	58.22	58.31	3.02	3.12	12.93	12.86
4_d-6_	o-ClC_6_H_4_	248	65	C_21_H_12_ON_4_ClBr	55.84	55.91	2.68	2.61	12.40	12.32
4_d-7_	p-ClC_6_H_4_	218	73	C_21_H_12_ON_4_ClBr	55.84	55.80	2.68	2.73	12.40	12.46
4_d-8_	p-OH,m-OCH_3_C_6_H_3_	226	78	C_23_H_18_O_4_N_4_Br	57.04	57.12	3.26	3.20	12.09	12.11

The antimicrobial screening of synthesized compounds was carried out by paper disc diffusion method[18] at 100 ppm against Gram +ve bacteria *B. substilis*, *S. aureus* and Gram –ve bacteria like *E. coli, P. vulgaris*. The antifungal activity of the compounds was assayed using fungal species *Aspergillus niger* and *Phytophora*. Standard antibacterial streptomycin and antifungal griseofulvin were also screened under similar condition for comparison. (Table 2)

**TABLE 2:** ANTIMICROBIAL SCREENING DATA OF THE DERIVATIVES OF 4

Comp	Bacteria			Fungi		
	*E. coli*	*p. valgaris*	*B. subtilis*	*S. aureus*	*Aspergillus niger*	*Phytophora spp*
4_a-1_	17	16	12	17	17	15
4_a-2_	5	7	15	14	5	11
4_a-3_	20	14	19	14	16	12
4_a-4_	7	10	7	4	8	1
4_a-5_	14	5	9	11	13	8
4_a-6_	16	20	17	15	17	20
4_a-7_	18	18	17	12	19	18
4_a-8_	12	15	11	12	12	14
4_b-1_	20	19	11	16	19	12
4_b-2_	4	8	9	7	4	8
4_b-3_	25	20	16	17	20	16
4_b-4_	9	4	14	4	9	7
4_b-5_	5	9	12	8	7	9
4_b-6_	17	11	16	11	19	19
4_b-7_	16	20	16	13	18	11
4_b-8_	11	12	14	11	12	12
4_c-1_	16	20	13	16	20	15
4_c-2_	9	4	8	4	8	10
4_c-3_	20	18	17	20	18	20
4_c-4_	8	6	12	6	8	5
4_c-5_	5	5	11	7	23	16
4_c-6_	22	19	18	17	23	16
4_c-7_	19	22	17	16	24	17
4_c-8_	11	11	7	12	9	12
4_d-1_	20	20	11	19	18	11
4_d-2_	14	14	6	8	12	7
4_d-3_	22	24	18	25	20	18
4_d-4_	7	8	11	5	7	7
4_d-5_	12	12	14	9	13	10
4_d-6_	25	23	20	16	24	20
4_d-7_	20	21	25	20	22	24
4_d-8_	15	12	14	11	15	11

Diameter of zone of inhibition in milimeters

The result indicated that some compounds exhibit good antimicrobial activity against the above mentioned bacterial and fungal species, while some compounds have moderate antimicrobial activity against both Gram +ve and Gram –ve bacterial and fungal species. It was absovered that introduction of ^-^OCH_3_ and ^-^Cl groups to the heterocyclic frame work enhanced antibacterial and antifungal activities.
